# Ambulance responses to older adults who have fallen: a systematic review

**DOI:** 10.1093/ageing/afaf228

**Published:** 2025-08-17

**Authors:** Imogen M Gunson, Chloé Barley, Andy Rosser, Laurna Bullock, Adam Lee Gordon, Tom Kingstone, Milica Bucknall

**Affiliations:** Faculty of Medicine & Health Sciences, Keele University, Newcastle-under-Lyme, UK; Research and Development, West Midlands Ambulance Service University NHS Foundation Trust, Brierley Hill, UK; Research and Development, West Midlands Ambulance Service University NHS Foundation Trust, Brierley Hill, UK; Research and Development, West Midlands Ambulance Service University NHS Foundation Trust, Brierley Hill, UK; School of Medicine, Keele University, Newcastle-under-Lyme, UK; Wolfson Institute of Population Health, Queen Mary University of London, London, UK; Academic Centre for Healthy Ageing, Barts Health NHS Trust, London, UK; Faculty of Medicine & Health Sciences, Keele University, Newcastle-under-Lyme, UK; Research and Innovation Department, Midlands Partnership NHS Foundation Trust, Stafford, UK; Faculty of Medicine & Health Sciences, Keele University, Newcastle-under-Lyme, UK

**Keywords:** older people, prehospital care, emergency medical response, accidental falls, systematic review

## Abstract

**Background:**

Approximately 10% of emergency ambulance calls are for adults, 65 years and older, who have fallen. Structured management of this group could improve outcomes and cost-effectiveness. This review sought to synthesise evidence for ambulance-based care of older adults who had fallen and the associated impact on patient outcomes.

**Methods:**

*Eligibility*. Peer-reviewed primary evidence, assessing older adults (aged ≥ 65) who had fallen and received an ambulance response.

*Information sources*. CINAHL (EBSCO), MEDLINE (Ovid), Embase (Ovid), HMIC (Ovid), Web of Science and AMBER were searched on 20 February 2025 with no date limit.

*Quality appraisal.* Joanna Briggs Institute’s critical appraisal tools.

*Synthesis.* PRISMA reporting, with narrative synthesis using Synthesis Without Meta-analysis guidelines.

**Results:**

Three thousand and forty-nine unique studies were identified. Nine studies were included, ranging from low- to high-quality randomised cluster trials and mixed-methods, prospective and retrospective cohort studies.

Patients whose care included fall-specific decision-making tools or referral pathways, were less likely to be conveyed to hospital and more likely to access alternative community healthcare. However, poor uptake of participating paramedics limits the impact on practice.

Few fall response or referral schemes were reported in the literature; those that exist have improved outcomes for patients. Where national practice guidelines exist, these were generally adhered to. Patient social characteristics, such as living alone, were the main reason for guideline deviation.

**Discussion:**

Where fall pathways were implemented, outcomes improved, though implementation is contextual. Ambulance staff adhere to guidelines, but whether the guidelines reflect current presentations and treatment opportunities is unclear. Further research is required to establish generalisable approaches.

## Key Points

- Ambulance calls for older adults who have fallen are a large workload, yet there is limited evidence on their responses.
- Interventional studies either did not meet recruitment targets or were resource intensive, so may have limited transferability.
- Ambulance staff generally adhere to guidelines; however, deviation is multifactorial, one of the factors being social considerations for the patients.
- Further research needs to be undertaken and published, to understand this growing population, for improving prehospital care.

## Introduction

A fall is ‘an event which results in a person coming to rest inadvertently on the ground … or other lower level’ [1]. A third of people aged 65 and older (≥65) fall each year globally [2]. Emergency calls following a fall comprised ~10% of ambulance service calls in England in 2022 [3], with similar figures in the USA [4] and Australia [5]. Falls were the leading cause of hospitalisation in England for those aged ≥ 65 in 2017/18 [6]. In addition to mortality, falls are associated with substantial morbidity, including loss in confidence and independence. People with mobility limitations reduce activity due to fear of falling, further reducing their mobility. This can have long-term implications in returning to independent living and quality of life [7–9].

The UK Falls Response Governance Framework acknowledges the increasing importance of falls for ambulance services [10], highlighting prevention, community resilience, effective telephone triage, referral and effective clinically safe fall responses as principles, aiming to avoid further harm and emphasising the focus on this area to understand how to improve care [11]. Internationally, approaches vary, such as prevention systems in Toronto, Canada, in which people who have fallen and called an emergency ambulance can be referred for rehabilitation and primary care [12] and coresponse services in London, England [3], and Queensland, Australia [5], where a paramedic and other health professionals respond to people who have fallen, accelerating assessment, treatment and intervention for incidents disproportionately impacting those aged ≥ 65.

Variation between nations is reflected in the lack of prehospital guidance within the World Falls Guidelines [13]. UK ambulance services consider ‘older adults’ as ≥65, although this heterogeneous population has varying risks of falling [14–16]. Internationally, guidelines suggest special considerations can start from 55 years, with considerable variation between providers [17]. Although Joint Royal Colleges Ambulance Liaison Committee (JRCALC) guidelines use age to guide decision-making [14], frailty is increasingly used within ambulance assessments and care pathway inclusion criteria. Frailty is a health state where inbuilt physiological reserves decline, leading to challenge when recovering from accidents (such as falls) and illnesses [18]. Many ambulance services have adopted the Clinical Frailty Scale as a judgement-based screening tool for frailty in people aged ≥ 65 [19].

Previous qualitative research found decision-making in prehospital care of older adults who have fallen to be complex and multifaceted [20, 21]. Staff in London, England, interviewed after testing a clinical assessment and referral flow chart tool, undertook phased decision-making that was both formal, based on structured assessment and clinical findings, and informal, impacted by their experience and the defensibility of their decisions [20]. Paramedics in Australia reported confidence in being protected by their employer and how much training they have undertaken influenced care provided [21]. A further Australian interview study found that lower acuity calls have more complex decision-making than if the patient is injured or has an acute episode, but a multitude of considerations are present across social, medical and financial situations [22]. There may be an assumption that by calling an ambulance a patient will be conveyed to hospital, leading to ambulance staff believing they are doing their job correctly or that safety outcomes will be better. The rationale for doing so is multiple but one contributor is staff’s fear of losing their job if they leave someone at home who goes on to have an adverse outcome. The NHS Staff Survey highlighted this, with <39% of ambulance staff respondents agreeing that their organisation treats staff who are involved in an error, near miss or incident fairly [23]. Additionally, some paramedics did not perceive attending uninjured older adults who fell as ‘real’ ambulance work, suggesting an underestimation of the importance and clinical priority of this patient group and a potential mismatch in paramedics’ professional identity and the calls they attend [21].

A UK study found emergency cases such as cardiac arrests are protocol driven, whereas urgent cases have more complex decision-making. Ambulance staff recognise the risks associated with patients waiting in overcrowded emergency departments and therefore try to use hospital-avoidance pathways, which can vary in provision by location, hours and skillset [24].

The published evidence on ambulance care provision to older adults who have fallen has not yet been synthesised. Undertaking this is important because prehospital healthcare providers are increasingly focussing on these calls, and synthesising the current evidence base could help inform these efforts.

The broad objectives for this synthesis were to examine the different response types, models or strategies used by ambulance services on calls for older adults who have fallen and outcomes for the population of adults when they have fallen.

### Review question

We intended to synthesise the evidence for ambulance care of older adults who had fallen and called an emergency ambulance (Population); compare different types of specialist ambulances and care delivered by nonspecialist ambulance personnel (Intervention); and assess morbidity, mortality, conveyance rates and other clinically relevant outcomes (Outcome).

## Methods

Methods and results are reported in line with the Preferred Reporting Items for Systematic Reviews and Meta-analysis (PRISMA) [25], using Synthesis Without Meta-analysis (SWiM) [26] guidelines for narrative synthesis.

The protocol was PROSPERO registered: CRD42023398342 [27].

### Eligibility criteria

#### Inclusion

Population: Older adults (≥65) who had low-velocity falls, such as from standing and received and emergency ambulance response.

Intervention: Articles reporting specialist ambulance fall responses or pathways, and standard emergency ambulance responses.

Outcome: Clinical findings or conveyance rates of the reported intervention.

Study designs: Full-text peer-reviewed primary evidence of any study design.

#### Exclusion

Undiscernible population age or inability to separate findings for those aged ≥ 65.

Non-English texts.

### Search strategy and selection criteria

On 20 February 2025 six databases were searched, using a structured predefined search strategy: CINAHL (EBSCO), MEDLINE (Ovid), Embase (Ovid), HMIC (Ovid), Web of Science and AMBER (UK ambulance repository). There was no retrospective date limit.

Three key concepts were included in our search strategy: older adults, ambulance response and falls (Appendix 1). The Boolean operator ‘AND’ combined these concepts, with MeSH subject headings mapped and exploded. Due to numerous variations in international terminology, multiple synonymous search terms were used to capture the breadth of studies. Manual searching of reference lists was undertaken for included full papers.

Database searches were performed, utilising Rayyan [28] to highlight duplicates, before manual review and removal (I.M.G.).

I.M.G. completed abstract screening; a second reviewer independently screened 10% of the abstracts (C.B.). All full texts were dual-screened for eligibility (I.M.G./C.B.).

A third reviewer (A.R.) resolved discrepancies at both screening stages.

### Data extraction

Data were extracted by two reviewers (I.M.G. and C.B./A.R.) into Microsoft Excel, aiding collation and comparison of variation.

The data items sought were aims/objectives, population characteristics, sample size, interventions, findings and outcomes: type of response, impact/outcome of response, decision-making with responses and outcomes of older adults who received a response. These are reported in Table 1 and Appendix 2.

**Table 1:** Included study characteristics.

Authors	Year	Country	Study design	Sample size
Snooks *et al.* [29]	2014	UK	Cluster randomised trial	42 paramedics 779 patients attended
Snooks *et al.* [30]	2017	UK	Cluster-randomised trial	215 paramedics 5914 eligible patients
Simpson *et al.* [31]	2014	Australia	Prospective observational cohort study	1720 cases submitted 110 excluded (10 duplicates, 55 lower priority, 5 < 65 years, 40 unlinkable) Final population 1610
Williams *et al.* [32]	2018	USA	Prospective cohort study	1473 eligible residents, 953 consented; 359 had 840 falls in 43 months
Simpson *et al.* [33]	2013	Australia	Retrospective descriptive analysis of routinely collected data	42 331 cases
Paul *et al.* [34]	2017	Australia	Retrospective population-based descriptive study	314 041 ambulance calls
Pyer *et al.* [35]	2015	UK	Mixed methods: Retrospective analysis of routinely collected data Survey and telephone interviews	12 119 cases pre-service introduction, 12 284 cases post-service introduction per year 294 survey respondents 16 service user telephone interviews
Nicholson *et al.* [36]	2022	UK	Mixed methods: Semistructured interviews Retrospective analysis of routinely collected data Focused review of 100 cases of conveyed patients	15 650 cases in retrospective analysis 100 in-depth case audits 10 interviews (8–12 were listed as intended sample)
Sheridan *et al.* [37]	2024	USA	Retrospective prevalence analysis of routinely collected data	1169 cases

### Quality appraisal, study and reporting risk of bias assessment

Two reviewers independently appraised the quality of each full text (I.M.G. and C.B./A.R.).

As this systematic review (SR) looks at varied methods for the prevalence of older adults who fall, and what response they receive, and does not seek to measure the effectiveness of these responses, Joanna Briggs Institute (JBI) critical appraisal tools (CAT) enabled study-specific assessment of methodological quality and the trustworthiness of results in published papers [38]. The Grading of Recommendations Assessment, Development, and Evaluation (GRADE) did not offer relevant approaches for this review with its focus on evidence and policy quality assessment; however, GRADE evidence profiles, provided in Online Supplementary Table 1, support interrogation of certainty [39].

Studies rated as low quality by both reviewers were excluded.

### Synthesis methods

Due to the heterogeneity of studies, a narrative synthesis was undertaken using SWiM [26].

The causes and levels of heterogeneity are reflected upon through reporting on the following:

- Methodology: randomised and nonrandomised studies.
- Statistics: variable information reporting.
- Clinical diversity: different intervention and outcome measures.

Data were extracted and presented in the included articles’ formatting style. Where data were missing, this is shown as ‘Not reported’.

Contextual mediating factors are reported, supporting evidence from diverse study designs. The focus was on effects and patterns, with an interpretation of the findings. Where published and applicable, effect size significance is reported in terms of the *P*-value or odds ratios (ORs) and 95% confidence intervals (CIs).

## Results

### Study selection

From six databases, 3499 studies were identified on 20 February 2025; following de-duplication 3049 studies were screened by title and abstract.

During title and abstract screening, inter-rater reliability was 98.69%, with Cohen’s *k* = 0.89. Both reviewers undertook independent full-text screening, reaching 96.75% agreement, with Cohen’s *k* = 0.82, again indicating very good agreement [40]. Conflicts were resolved by the third reviewer; this resulted in 12 included texts [25].

One study was represented twice within full texts, the journal article and the Health Technology Assessment report, which was excluded from data extraction, to prevent duplication of reported results [41].

**Figure 1:**
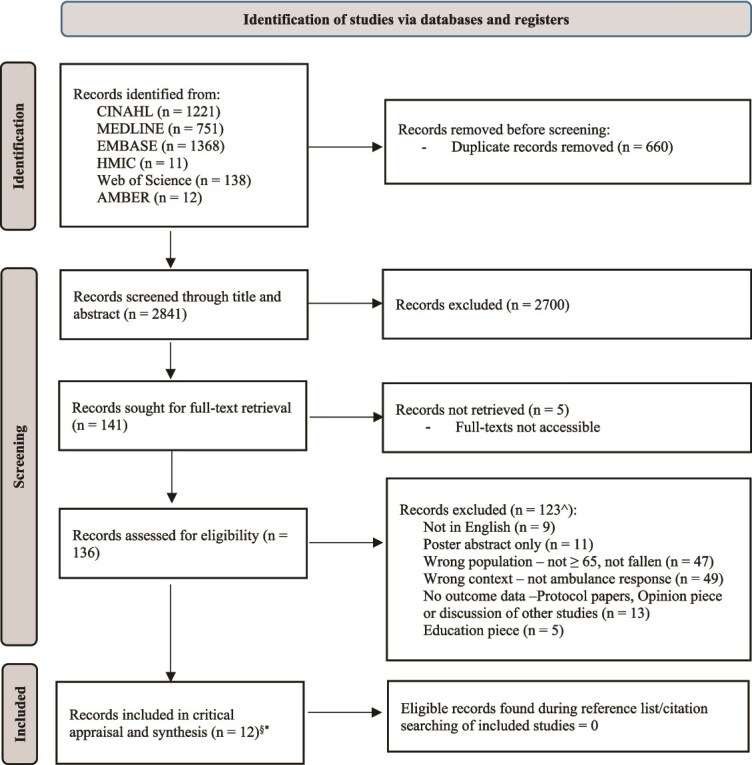
PRISMA flow diagram [25]. ^ Several studies were identified as excluded for both wrong population and context. ^§^Two excluded from synthesis following quality appraisal. ^*^One study (SAFER2) has both a journal article [30] and a Health Technology Assessment report [41]; data only extracted from journal article focussed on the intervention.

Eleven texts underwent critical appraisal, following which two studies were excluded due to concerns for data assumptions and interpretation, rather than evidenced findings (Figure 1). The included studies (Table 1) were from the UK (*n* = 4), Australia (*n* = 3) and the USA (*n* = 2), with cohort studies [31–34, 37], randomised cluster trials [29, 30] and mixed-methods studies (interviews, surveys and retrospective case reviews) [35, 36]. See Appendix 2 for further information on the included studies.

### Ambulance responses

Eight studies provided characteristics of those who had fallen, alongside the response and outcome of emergency calls [29–34, 36, 37].

Simpson *et al.*’s prospective cohort study conducted in New South Wales (NSW), Australia [33], found increased likelihood of falling and being conveyed to hospital for the oldest-old compared with the youngest-old, with no difference in injurious falls between the youngest-old and oldest-old; however, females were more likely to have injuries reported. The youngest-old were defined as aged 65–74 years, middle-old as 75–84 years and oldest-old as ≥85 years [42]. Nicholson’s UK study found that frailty and living alone were more influential in paramedics’ conveyance decisions than guidelines [36]. Limited patient outcomes were reported in this observational study, only the appropriateness of conveyance guidelines. Appropriate conveyance is where patients are conveyed to hospital as the place most suitable for their healthcare needs; inappropriate conveyance is when patients could safely and efficiently be cared for in the community, if sufficient care pathways exist [43].

Paul *et al.*’s retrospective observational study reported characteristics and demographics of patients aged ≥ 65 who called an ambulance after falling in NSW, Australia, between 2006 and 2013 with similar figures to those of Simpson *et al.*, where half the conveyed patients had sustained an injury—mainly limb injuries and suspected fractures [33, 34]. Impact is reduced due to both studies having incomplete reporting of injuries and lacking linked outcome data. Simpson’s later study found that most conveyed patients had either an injury or abnormal vital signs. For those conveyed, an increased fall risk was found following discharge due to poor guideline adherence of preventative recommendations [31].

The Crisis Response Falls Service (CRS) operated in Northamptonshire, UK, combining an ambulance crew with specialist lifting equipment and enhanced patient diagnosis training and a multidisciplinary social care team who assessed people at home to identify future fall risks, while providing mobility aids [44]. They identified health and social care needs, enabling people to live independently and reduce repeat falls [35]. They referred directly to geriatricians and community nursing teams, and trained together to understand each other’s roles. Significant reductions in all hospital conveyance for falls 12 months after CRS introduction, compared to the 12 months before (*P* = .028), were found. Hospital conveyance usually occurred overnight when the CRS did not operate. Simpson also found that conveyance was more likely out of hours [OR 1.06 (95% CI 1.0–1.1)] [33]. Pyer *et al.* were unable to differentiate between specialist fall ambulances and standard ambulances within the routine data; therefore, inferences were based on comparisons between CRS operating hours (7 a.m.–5 p.m.) when conveyances reduced. Linked outcomes to ascertain whether avoiding conveyance led to improved long-term patient outcomes were not reported [35].

Sheridan retrospectively assessed 9.5 years of activations for falls in a suburban US system [37]. They differentiated between lift assists, i.e. returning the patient to a position of safety without conveyance, and falls, i.e. unintentional descents. Overall most responses were to females (55.2%, *P* = <.001), with 60% conveyed (*P* ≤ .001), and patients aged 85+ (61.2%); however, age had no relationship to conveyance to hospital (*P* = .442). Most calls were between 06:00 and 17:59 (*P* = .002) rather than between 18:00 and 05:59, with 77.8% conveyed compared to 6.5% of lift assists.

### Decision-making support tools

Three studies assessed decision-making with tools or pathways [29, 30, 32]. Williams *et al.* introduced a service in Wake County, NC, USA, in one of 22 assisted living facilities, for a primary care support tool following ground-level falls [32]. Paramedics assigned patients following a decision-making tool—tier 1: recommended conveyance; tier 2: discuss with physician; and tier-3: do not convey. Over half the tier-1 patients were deemed to be time-critical for emergency conveyance, with all but one patient accepting the recommendation. Tier 3 had the most nonconveyed patients, in line with the guidance. However, four patients were deemed to be time-critical by staff for reasons beyond the fall, and 4% of all tier-3 patients requested conveyance. Tier 2 (*n* = 239) experienced 20% conveyance, half of whom were time-critical. Most patients received appropriate care, suggesting guideline adherence and multidisciplinary collaboration provided safe and effective decision-making. Combining ambulance staff assessment with primary care physician support suggests good communication and shared decision-making could improve patient outcome and experience [32]. Quality was moderate, due to confounders being reported in the data but not explicitly identified, raising the question of how they managed them.

Support and Assessment for Fall Emergency Referrals (SAFER) provided decision-making tools that guided ambulance staff on which patients were appropriate for a fall service referral and which should be conveyed, through integrating vital signs and environmental risks [29]. Although these tools appeared safe and effective, this trial was underpowered, with an unmet recruitment target; therefore, caution was needed when interpreting the results. The SAFER trial assessed the Computerised Clinical Decision Support (CCDS) tool for nonfall specialist ambulance staff in two UK ambulance services, finding double the fall service referrals (OR 2.04, 95% CI 1.12–3.72), with only 9 minutes of scene time extensions (95% CI 2.32–15.26). There was no difference between the arms for harm, satisfaction or quality of life outcomes [29]. Being an underpowered study, true potential benefits may not have been found; however, the report was deemed to be of high quality due to data transparency. Qualitative work into the complexity of decision-making and tool use in SAFER found it did not assist with decision-making as it was deemed too basic. Ambulance staff felt their experience and clinical judgement skills were superior, as decision-making is their primary skill [45].

The CRS reduced hospital admissions and adverse events similarly to SAFER. Uptake was limited by nonspecialist ambulance responders, but good from fall specialists [35]. UK studies struggled to recruit and maintain participants [30, 35]. Contrastingly, the North American study assessing collaborative decision-making between paramedics (nonfall specialists) and primary care physicians for appropriate transport decisions was highly resource intensive [32], which may explain the effectiveness but limits transferability to the UK and other settings.

### Guidelines and training for conveyance and pathway referrals

SAFER2 was undertaken to assess through a cluster randomised design how an education and pathway programme impacted the conveyance decisions of non-specialist ambulance responders and the outcomes. Improvements in care were noted; however, as with the earlier study recruitment targets were not met [30]. In SAFER2, interpersonal skills were rated higher in the intervention arm for staff who had participated in the education, and patients who had fallen recontacted 999 fewer times within the following 6 months from intervention paramedic attendance (adjusted OR = 0.899, 95% CI 0.799–1.011), although this finding was insignificant [30]. However, both study arms had two-thirds of patients experiencing further emergency episodes or death by 6 months. This study reported 80% of the primary outcome data, which is higher than many studies undertaking linked follow-up of health records, strengthening the confidence in its findings for the limited number of patients enrolled. Repeat ambulance calls for falls were reduced when patients received fall referrals as part of their ambulance service care, and there were improved patient outcomes and good clinician satisfaction when referral pathways and protocols were followed [30, 32, 35].

The NHS’s Planning to Safely Reduce Avoidable Conveyance guidance development will review guideline adherence or development of guidelines if absent [43]. One study assessed the adherence to guidelines. Nicholson *et al.* studied this for nonfall specialist ambulances, in the subset of head-injured older adults [36]. Using descriptive statistics, they reported that most patients are treated in accordance with guidance, but on interviewing ambulance staff it was discovered that the staff’s decision-making was impacted by social situations, leading to deviations from guidelines.

### Outcomes for the patient population: do they differ depending on the response?

Seven studies reported outcomes, with their quality ranging from low to moderate to high [29–35].

Patients surveyed following CRS responses reported improved experiences with reduced conveyance [35]. Staff experience was not assessed for ease of use or application of eligibility criteria, nor why it was utilised more by the specialist fall responders compared to the standard response. Although seemingly safe and cost-effective, underrecruitment to the CCDS tool and the education programme renders the impact of the interventions unable to be reliably assessed for implementation into practice [29, 30].

The American study of primary care physicians and paramedics discussing conveyance decisions dependent on tool outcome found a positive impact on patients. It had good adherence for patients meeting conveyance criteria being taken to hospital unless they refused. One-fifth of patients requiring discussion with a primary care physician were conveyed to hospital; therefore, nearly 80% remained at home [32]. A small number of those deemed not to require conveyance were taken due to patient request.

### Certainty of evidence

The study quality appraisals ranged from low to high, with 11 recommendations for inclusion, and 2 recommended exclusion due to methodological concerns (Table 2). Following discussions among the independent reviewers, the quality was assessed using the most appropriate study-type-specific JBI tools with narrative synthesis. There are variations between these tools to account for differences in study designs.

**Table 2:** Quality appraisal and inclusion decision.

	Quality?	Include	JBI tool
Snooks *et al.* [29]	High	Yes	JBI Critical Appraisal Tool for assessment of risk of bias for randomised controlled trials [46]
Snooks *et al.* [30]	High	Yes	JBI Critical Appraisal Tool for assessment of risk of bias for randomised controlled trials [46]
Simpson *et al.* [31]	Moderate—lack of clarity on why routine data couldn’t be used leading to potential bias	Yes	JBI Critical Appraisal Checklist for studies reporting prevalence data [47]
Williams *et al.* [32]	Moderate to high—confounders reported but not explicitly labelled as such	Yes	JBI Critical Appraisal Checklist for cohort studies [48]
Simpson *et al.* [33]	Moderate to high—lacked detail on dealing with missing data	Yes	JBI Critical Appraisal Checklist for studies reporting prevalence data [47]
Paul *et al.* [34]	High	Yes	JBI Critical Appraisal Checklist for studies reporting prevalence data [47]
Pyer *et al.* [35]	Low to moderate—results not clearly presented in the article and lack of researcher influence statements	Yes	JBI Critical Appraisal Checklist for cohort studies [48]
Nicholson *et al.* [36]	Moderate—unclear representation of participant voices and lack of researcher influence statements	Yes	JBI Critical Appraisal Checklist for Qualitative Research [49] and JBI Critical Appraisal Checklist for studies reporting prevalence data [47]
Sheridan *et al.* [37]	High	Yes	JBI Critical Appraisal Checklist for studies reporting prevalence data [47]
Hutchinson *et al.* [50]	Low—concludes the protocol to be effective, yet almost half of the patients recommended nonconveyance were admitted due to missed or misinterpreted assessments, with two going on to die within 30 days. No report on why the authors believe the nonconveyance protocol to be feasible	No	JBI Critical Appraisal Checklist for diagnostic test accuracy studies [51]
Darnell *et al.* [52]	Low—lack of detail in methods for congruity between methodology and results.	No	JBI Critical Appraisal Checklist for Qualitative Research [49]

Bias risk was prevalent across studies, due to perceived publication reporting limitations; therefore, caution must be taken with the conclusions (Table 3). Unanimous decisions for each paper were received. Low to high certainty of evidence for the outcomes was found, due to the aforementioned concerns around reporting limitations (Table 4). Further assessment of certainty is provided in Online Supplementary Material 1 with the GRADE evidence profiling.

**Table 3:** Risks of bias.

	Allocation concealment (selection bias)	Selective reporting (reporting bias)	Incomplete outcome data (attrition bias)	Blinding of participants (performance bias)	Other risks of bias
Snooks *et al.* [30]	Bias potential	Unclear	Bias potential	Bias potential	Unbiased
Williams *et al.* [32]	Bias potential	Bias potential	Unbiased	Bias potential	Unclear
Simpson *et al.* [31]	Bias potential	Unclear	Bias potential	Bias potential	Unclear
Snooks *et al.* [29]	Bias potential	Unbiased	Bias potential	Bias potential	Unbiased
Paul *et al.* [34]	N/A	Unclear	Unbiased	N/A	Unclear
Simpson *et al.* [33]	N/A	Unbiased	Unclear	N/A	Bias potential
Nicholson *et al.* [36]	N/A	Unclear	Unbiased	N/A	Bias potential
Pyer *et al.* [35]	N/A	Unclear	Unclear	N/A	Bias potential
Sheridan *et al.* [37]	N/A	Unclear	Unbiased	N/A	Bias potential

**Table 4:** Certainty of evidence.

Outcome	Confidence in body of evidence
Type of response: standard emergency medical service/ambulance, specialist falls, referral pathway	High certainty with recording objective and well documented in the studies [29, 30, 32, 33, 35–37] Total number of participants: 79 287 Study types: cluster-randomised trials, prospective cohort studies, retrospective descriptive study, mixed-methods studies—retrospective routinely collected data analysis, surveys and interviews
Response impact or outcome	Medium certainty due to missing data and heterogeneity of outcomes [29, 30, 32, 35, 37] Total number of participants: 21 296 Study types: cluster-randomised trials, prospective cohort studies, mixed-methods studies—retrospective routinely collected data analysis, surveys and interviews
Decision-making	Medium certainty due to range of quality, and missing data in the studies [30, 32, 36] Total number of participants: 22 414 Study types: cluster-randomised trial, prospective cohort study, mixed-methods studies—retrospective routinely collected data analysis and interviews
Outcomes of older adults who have fallen and received an ambulance response	Low certainty due to substantial heterogeneity. All the studies reported outcomes; however, this definition varied between conveyance, through to 12 months [29–37] Total number of participants: 394 938 Study types: cluster-randomised trials, prospective cohort studies, retrospective descriptive studies, mixed-methods studies—retrospective routinely collected data analysis, surveys and interviews

## Discussion

The key findings of this systematic review were the importance of referral pathways, decision-making tools, training to improve patient care through fewer inappropriate hospital conveyances, better guideline adherence, increased specialist fall service referrals and improved patient satisfaction. Generalisability is limited by resource intensiveness or low clinician study uptake. However, if resourcing could be balanced, the North American study suggests a positive healthcare delivery impact potential, and if implemented could potentially sustain the high level of primary care physician interaction with paramedics. Ambulance staff voices are poorly represented, making implementation and uptake issues difficult to understand.

There is evidence that ambulance staff adhere to guidelines; however, it is unclear whether the guidelines are fully reflective of the current presentations and treatment opportunities in different localities. Without this clarity, the guidelines may be ineffective in managing the populations now presenting to ambulance services.

Patient outcomes were lacking detail, with underpowered studies and little data linkage providing longitudinal patient outcomes. Patient-reported outcomes were also lacking for how they feel about specialist fall responses or nonspecialist ambulances using tools and pathways to support their care provision. The JRCALC guidelines emphasise the importance of shared decision-making; therefore, the omission of this within published data highlights a crucial gap in evidence [14].

The strengths of this review are that it is the first to report on ambulance responses across specialist resource and tool use in older adults, independent undertaking of screening and data extraction and adherence to the published protocol [27] with strong inter-rater reliability.

The limitations stem from the available data being heterogeneous, leading to purely narrative synthesis of evidence. The evidence quality proved varied, from low to high, limiting the transferability and comparison of findings. Variation across international systems furthers heterogeneity, with differing response models and skillsets included as eligible responders. With the variety of study types anticipated and identified, critical appraisal was undertaken with JBI CAT for each method. As this review sought to systematically assess evidence, rather than guide policy development, this is still a valid critique.

Inconsistency in classifying older adults rendered interpretation of the existing literature challenging and introduces the question of whether age is the best way to triage patients into specific services. An emerging literature on young frailty recognises intersectional risk factors for frailty, including social isolation, lifestyle factors, ethnicity and long-term conditions—perhaps in the near future age will not be a necessary criterion for access to frailty-specific services [53].

Due to the challenge of blinded randomised controlled trial testing pathways, this design risks selection bias for when clinicians enact the pathway, as it not possible for them to be blinded for this. Retrospective routine data studies enable observational findings for this patient population; however, they have a tendency to miss high amounts of data and they cannot assess unreported confounders. Full texts were only included if the studies were published in English, due to reviewer linguistic limitations. Through excluding eight articles (<6% of 136) at full-text screening, across six languages (Japanese *n* = 3, Turkish *n* = 1, Portuguese *n* = 1, Spanish *n* = 1, German *n* = 1 and Swedish *n* = 1), there is a risk of missing valuable themes and findings. Future work should look to include translation facilities, should resources be available.

In conclusion, this systematic review finds limited published reports on the ambulance response to older adults who have fallen, despite the volume of cases within emergency ambulance service workloads. Where decision-making tools and fall pathways are implemented, patient care improves. However, work is required to improve ambulance staff’s use of fall tools or pathways.

## Supplementary Material

aa-25-0927-File002_afaf228

aa-25-0927-File003_afaf228
