# Supplementary material for: Ambulance responses to older adults who have fallen: a systematic review
Source: Age Ageing. 2025 Aug 17;54(8):afaf228. doi: 10.1093/ageing/afaf228 (PMC12358043; doi:10.1093/ageing/afaf228)
Supplement: aa-25-0927-File002_afaf228 [file aa-25-0927-file002_afaf228.docx]

**ONLINE SUPPLEMENTARY TABLE 1 – GRADE Evidence Profiles**

This GRADE table has been provided by the authors to assist readers in interpreting the results for quality of key outcomes.

| ***Outcome*** | ***Number of studies (Design)*** | ***Limitations*** | ***Inconsistency*** | ***Indirectness*** | ***Imprecision*** | ***Publication bias*** | ***GRADE Certainty of evidence*** |
| --- | --- | --- | --- | --- | --- | --- | --- |
| Type of ambulance response | 7 (CRTs, Prospective cohort studies, Retrospective descriptive study, Mixed-Methods) | High potential with retrospective and descriptive studies lacking confounder control | No evidence | Potential – imprecision with retrospective routine data | High potential – smaller sample sizes | No evidence | Low |
| Response impact or outcome. | 5 (CRTs, Prospective cohort studies, Mixed-Methods) | Potential – non-randomised and retrospective studies included | No evidence | Potential – imprecision with retrospective routine data | Potential | No evidence | Moderate |
| Decision-making support tools | 3 (CRT, Prospective cohort study, Mixed-Methods) | Moderate potential – CRT lower risk but cluster bias potential | No evidence | Potential – mixed-methods and descriptive studies | No evidence – large sample sizes | No evidence | Moderate |
| Outcomes of older adults who have fallen and receive an ambulance response. | 9 (CRTs, Prospective cohort studies, Retrospective descriptive studies, Mixed-Methods) | Potential – non-randomised and retrospective studies included | No evidence | Potential – mixed-methods and descriptive studies | No evidence – large sample sizes | No evidence | Moderate |

*Abbreviations*: GRADE - Grading of Recommendations Assessment, Development and Evaluation; CRT – Cluster Randomised Trial

**GRADE Working Group.** Grading quality of evidence and strength of recommendations. BMJ 2004; 328(7454):1490.
