# Supplementary material for: Ambulance responses to older adults who have fallen: a systematic review
Source: Age Ageing. 2025 Aug 17;54(8):afaf228. doi: 10.1093/ageing/afaf228 (PMC12358043; doi:10.1093/ageing/afaf228)
Supplement: aa-25-0927-File003_afaf228 [file aa-25-0927-file003_afaf228.docx]

# The Ambulance Responses to Older Adults who have Fallen: A Systematic Review

# AppendIx 1

MEDLINE search strategy

| 1 | exp Aged/ | 3644640 |
| --- | --- | --- |
| 2 | Aging/ | 261205 |
| 3 | (old* adj2 (adult* or age* or people or person or men or women)).ti,ab,kf. | 374957 |
| 4 | ageing.ti,ab,kf. | 62295 |
| 5 | elder*.ti,ab,kf. | 334539 |
| 6 | Frail*.ti,ab,kf. | 43951 |
| 7 | Frailty/ | 12564 |
| 8 | 1 or 2 or 3 or 4 or 5 or 6 or 7 | 4057492 |
| 9 | Accidental Falls/ | 29620 |
| 10 | (fall or falls or fallen or faller or falling or fell).ti,ab,kf. | 292816 |
| 11 | (slip or slipped or slips or slipping).ti,ab,kf. | 21269 |
| 12 | (trip or tripped or trips).ti,ab,kf. | 13653 |
| 13 | 9 or 10 or 11 or 12 | 331539 |

**Appendix 2**

Included study aims, population characteristics, and outcomes of older adults who have fallen and receive an ambulance response

| *Authors* | *Aims/Objectives* | *Population characteristics* | *Outcome measure* |
| --- | --- | --- | --- |
| Snooks et al.​[39] | To evaluate effectiveness, safety and cost-effectiveness of Computerised Clinical Decision Support (CCDS) for paramedics attending older people who fall. | Paramedics - working at two ambulance stations with a fall pathway within two UK ambulance services.  Patients - 65 or older, within catchment of falls service (excluding nursing home residents), attended by study paramedic, with first 999 call for a fall.    Patient characteristics in intervention and control:   \| Female \| 65% and 61% \| \| --- \| --- \| \| Median age (inter quartile range) \| 83 (77–89) and 82 (76–88) \| | 1. Effectiveness – proportion of participants left at scene without conveyance to an Emergency Department and proportion referred to falls services. 2. Safety – proportion of participants with adverse events up to 1 month after the initial call (999 call, Emergency Department attendance, emergency admission to hospital, or death).   Cost-effectiveness – comparison of costs of implementation of  CCDS for paramedics and its benefits in the form of patient utility modelled over 12 months. |
| Williams et al.​[33] | To determine whether unnecessary transport to hospital can be avoided following a ground-level fall in older adults. | Convenience sample of residents in 22 assisted living facilities served by 1 group of primary care physicians.  Mean age: 86 years; 76% female. | Primary outcome: Subsequent emergency events within 1 and 6 months of recruitment (death, emergency admissions, Emergency Department (ED) attendances, and 999 calls).  Secondary outcomes:  - at initial event - disposition (conveyed to the ED, referred to falls service, or left at scene with no referral), clinical documentation completion rates, duration of ambulance service job cycle (from the call to time when the ambulance was free for the next call), and episode of care (from the call to the time when the patient was left at home, discharged from the ED, or admitted to the hospital).  - at 1 and 6 months after initial event - self-reported further falls, further fractures, days spent in the hospital, health-related quality of life according to the 12-Item Short-Form Health Survey, “fear of falling” according to the modified Falls Efficacy Scale, and costs of care.  - at 1 month after initial event - patient satisfaction according to the Quality of Care Monitor. |
| Simpson et al.​[34] | To quantify the size and scope of the operational burden for a large ambulance service arising from older people who have fallen and to describe this population. | Emergency calls to patients aged 65 years and older for falls in New South Wales, Australia - excluding cases which appeared to have an underlying medical cause or trauma caused by external forces acting upon the patient, according to treatment recorded by paramedics.  The median age of patients was 83 (interquartile range 76–87), with 62% female. | Size and scope of operational burden of fallers on the ambulance service - Disposition, temporal patterns (time of day, day of week, season/month), geographical location of call, age of patient, sex, type of trauma (if any) sustained by fall, type of response allocated (urgent vs non urgent), presenting physiology. |
| Snooks et al.​[38] | To assess the benefits and NHS costs of a complex intervention comprising education, clinical protocol, and pathway, enabling paramedics to assess older people after a fall and refer them to community-based falls services when appropriate. | Adults over 65 years old who were attended by a trial paramedic following a 999 call coded as “fall” without any priority symptoms. Participants must have been within catchment area of existing falls service.  4,704 (80%) patients consented for follow-up.  2,420 in the intervention group and 2,284 in the control group.  Mean age: 82 years - 82.54 intervention, 82.14 control  Female - 61.2% intervention, 64.7% control group  41.8% of calls were out of hours in both arms | The primary outcome was subsequent emergency events within 1 and 6 months of recruitment (death, emergency admissions, Emergency Department (ED) attendances, and 999 calls).  Secondary outcomes at the index event were disposal (conveyed to the ED, referred to falls service, or left at scene with no referral), clinical documentation completion rates, duration of ambulance service job cycle (from the call to time when the ambulance was free for the next call), and episode of care (from the call to the time when the patient was left at home, discharged from the ED, or admitted to the hospital).  Secondary outcomes at 1 and 6 months after index event were self-reported further falls, further fractures, days spent in the hospital, health related quality of life according to the 12-Item Short-Form Health Survey, “fear of falling” according to the modified Falls Efficacy Scale, and costs of care. |
| Paul et al.​[36] | To describe characteristics and temporal trends of fall-related ambulance service use and hospital admission in older adults in New South Wales, Australia. | People aged 65 years and older who requested an ambulance in New South Wales, Australia for a fall-related incident between 2006 and 2013.    Female 61.5%   \| Age: \| 65-74 19.5%  75-84 39.9%  85+ 40.6% \| \| --- \| --- \| | As a result of fall: Transport vs non-transport; Injury/no injury.  Characteristics and demographics of patients who call for EMS assistance following a fall. |
| Simpson et al.​[37] | To describe:   1) the characteristics of older people who fall and call an ambulance  2) the characteristics of the falls and the ambulance response they receive. | Female 61%  Community dwelling 82%  First time calls for falls 42%, fall call within last month 15%  Median response time was 15 minutes (IQR 10-24)  Reasons for falls: Loss of balance (30%) and Simple trips (25%).  New injury or pain 73%  Not transported 28% | 24 variables (plus data from EPR/CAD) including:  Age, Sex, non-English speaking, residential status, ambulatory status, number of prescription and anticoagulant medicines. Personal alarm system; falls in last 12 months (yes/no); number of falls in 12 months; time on floor (mins); on floor on ambulance arrival (yes/no); place of fall; fall from (standing/chair etc); location of fall (in residence); reason for fall; change in function post fall; physiological status; injury/pain sustained; location and type of injury;  HR, RR, SBP, SP02 and AVPU |
| Nicholson et al.​[40] | This study used routine data and semi structured interviews to explore the factors that influence paramedic decision-making when considering whether to convey an adult aged 65 years and over with a minor head injury to the Emergency Department (ED).  It also assesses adherence to current National Institute for Health and Care Excellence (NICE) and Joint Royal Colleges Ambulance Liaison Committee (JRCALC) guidelines. It may inform the development of future interventions to reduce avoidable conveyance. | Routine data population (Patients aged 65 and over with Head Injury): Average patient age was 83 years. 53% female.  70.5% were conveyed to ED, and 1.6% were taken to other places (e.g. hospice, MIU, community hospital)    Interview population: Operational paramedics, 70% male. Years experience as a qualified paramedic ranged from 1.5 years to 27 years.  Participants roles included: Paramedic; Newly Qualified Paramedic (NQP); Specialist Paramedic; and Remote Clinical Validation. | Prevalence of conveyance in routine data analysis.  100 case in-depth audit - proportion of conveyed cases which meet NICE and JRCALC Head Injury (HI) guidance for ED referral.    Interviews were inductive and phenomenological therefore no outcomes were predefined. The topic guide provides a clear steer on the intentions of interviews - defining minor HI, experience of this patient group and convey/non convey decisions, factors influencing decision making, ease of identifying safe non conveyance older adult, barriers to non-conveyance, identifiers of safe non-conveyance, available alternative pathways, use of guidelines and tools. |
| Pyer et al.​[41 | The aim of the study was to identify the effectiveness of the service, with particular emphasis on patient experience and service impact. | Recipients of care from Crisis Response Service (CRS).  Most survey respondents were over 75 years (84%).  75% of referrals were single instances, 18.8% had 2 referrals.  43% of referrals were from specialist falls ambulances, with 49% made to avoid hospital admission.  Demographics of patients attended not reported. | Patient experience of the service.   Number of falls related calls to ambulance service pre and post CRS opening.  Conveyance rates pre and post CRS.  Cost-benefit of CRS. |
| Sheridan, Wiseman and Quatman​[35] | Identify time of day EMS are called for falls or lift assists of patients aged 65+. Identify transport patterns of these calls. | Older adults (65+) who have fallen and received EMS response. | Time of day. Conveyance rate |

# Abbreviations:

AVPU – alertness scale (Alert, Voice, Pain, Unresponsive); CAD – Computer Aided Dispatch; CCDS - Computerised Clinical Decision Support; CRS - Crisis Response Service; ED – Emergency Department; EMS – Emergency Medical Services; EPR – Electronic Patient Record; HI – Head Injury; HR – Heart Rate; JRCALC - Joint Royal Colleges Ambulance Liaison Committee; MIU – Minor Injuries Unit; NICE - National Institute for Health and Care Excellence; NQP - Newly Qualified Paramedic; RR – Respiratory Rate; SBP – Systolic Blood Pressure; SP02 – Oxygen saturation
